# Cross-sectoral inter-rater reliability of the clinical frailty scale – a Danish translation and validation study

**DOI:** 10.1186/s12877-020-01850-y

**Published:** 2020-11-03

**Authors:** Søren Kabell Nissen, Anders Fournaise, Jørgen T. Lauridsen, Jesper Ryg, Christian H. Nickel, Claire Gudex, Mikkel Brabrand, Karen Andersen-Ranberg

**Affiliations:** 1 Institute of Regional Health Research, Centre South West Jutland, University of Southern Denmark, 6700 Esbjerg, Denmark; 2 Department of Emergency Medicine, Hospital of South West Jutland, 5700 Esbjerg, Denmark; 3 Department of Cross-sectoral Collaboration, Region of Southern Denmark, 7100 Vejle, Denmark; 4 Department of Geriatric Medicine, Odense University Hospital, 5000 Odense, Denmark; 5 Epidemiology, Biostatistics and Biodemography, Department of Public Health, University of Southern Denmark, 5000 Odense, Denmark; 6 Department of Business and Economics, University of Southern Denmark, 5230 Odense, Denmark; 7 Department of Clinical Research, University of Southern Denmark, 5000 Odense, Denmark; 8 Emergency Department, University Hospital Basel, University of Basel, 4031 Basel, Switzerland; 9 Open Patient data Explorative Network (OPEN), Region of Southern Denmark, 5000 Odense, Denmark

**Keywords:** Cross-sectoral collaboration, Continuity of care, ISPOR translation, Validation, Geriatrics, Clinical frailty scale, MESH-terms, Reliability, Interobserver, Frailty, Older people, EMBASE: Interrater reliability

## Abstract

**Background:**

Focus on frailty status has become increasingly important when determining care plans within and across health care sectors. A standardized frailty measure applicable for both primary and secondary health care sectors is needed to provide a common reference point. The aim of this study was to translate the Clinical Frailty Scale (CFS) into Danish (CFS-DK) and test inter-rater reliability for key health care professionals in the primary and secondary sectors using the CFS-DK.

**Methods:**

The Clinical Frailty Scale was translated into Danish using the ISPOR principles for translation and cultural adaptation that included forward and back translation, review by the original developer, and cognitive debriefing. For the validation exercise, 40 participants were asked to rate 15 clinical case vignettes using the CFS-DK. The raters were distributed across several health care professions: primary care physicians (*n* = 10), community nurses (n = 10), hospital doctors from internal medicine (*n* = 10) and intensive care (*n* = 10). Inter-rater reliability was assessed using intraclass correlation coefficients (ICC), and sensitivity analysis was performed using multilevel random effects linear regression.

**Results:**

The Clinical Frailty Scale was translated and culturally adapted into Danish and is presented in this paper in its final form. Inter-rater reliability in the four professional groups ranged from ICC 0.81 to 0.90. Sensitivity analysis showed no significant impact of professional group or length of clinical experience. The health care professionals considered the CFS-DK to be relevant for their own area of work and for cross-sectoral collaboration.

**Conclusion:**

The Clinical Frailty Scale was translated and culturally adapted into Danish. The inter-rater reliability was high in all four groups of health care professionals involved in cross-sectoral collaborations. However, the use of case vignettes may reduce the generalizability of the reliability findings to real-life settings. The CFS has the potential to serve as a common reference tool when treating and rehabilitating older patients.

**Supplementary Information:**

The online version contains supplementary material available at 10.1186/s12877-020-01850-y.

## Background

It is a global concern that even highly effective health care systems will struggle to meet the demands of the increasing share of aged and frail populations [1]. Frailty is a health state associated with the ageing process and is recognized as a good estimate of changes associated with molecular ageing, i.e. biological age [2, 3]. Frail citizens often need support from several different health and social care providers and are frequently subject to fragmented continuity of care due to poor cross-sectoral coordination and communication [4]. Knowledge of frailty status enables the identification of citizens who need tailored treatment and care plans within and across health care sectors [5]. This requires the primary and secondary health care sectors to use a standardized frailty measurement tool that has transdisciplinary acceptance [6, 7]. Such a tool could act as a reference point for treatment and care and serve as a safeguard against ageism in allocation of healthcare resources.

Multiple scales and instruments for measuring frailty exist and have been tested in various settings [5, 8]. The Frailty Index [9] and the Frailty Phenotype [2] are the most prominent and are often used as reference. However, the Clinical Frailty Scale (CFS) is increasingly used in clinical research across medical specialities and emergency medical services [5], likely due to its ease of use and speed of completion [10].

**Fig. 1:**
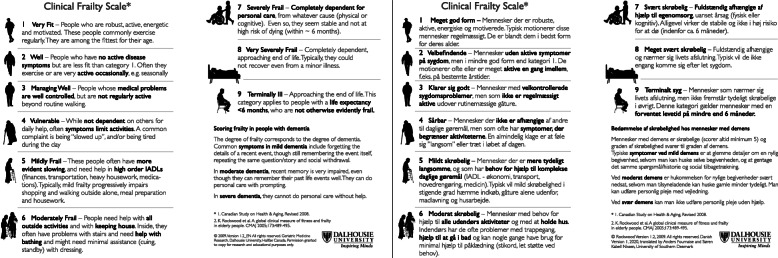
The Clinical Frailty Scale source instrument in English (left) and the Danish translation (right). IADL = Instrumental Activities of Daily Life. Printed with permission from copyright holder [9]

The CFS was developed in Canada in 2005 and was validated for diagnostic accuracy of frailty in people aged 65 years and over in the primary care sector [9]. The original 7-point version was modified in 2008 by the developers to its current form, a 9-point scale with pictograms (Fig. 1) [11]. Since then, predictive performance has also been validated in the secondary health care sector for multiple outcomes, including mortality and admissions to intensive care [12], length of stay [13], outcomes from resuscitation [14], interventions in the intensive care unit [15], and survival in the intensive care unit [16]. Although the CFS is of particular interest for cross-sectoral implementation, its psychometric properties such as inter-rater reliability have not been evaluated or compared across the primary and secondary health care sectors, which is a necessity for the establishment of a common reference for frailty measure.

The aim of the current study was first to translate the CFS into Danish (CFS-DK) using standard methodology and then to test inter-rater reliability for key health care professionals in the primary and secondary sectors using the CFS-DK.

## Methods

The study was conducted from 16th March to 8th May 2020.

### Translation process

**Fig. 2:**
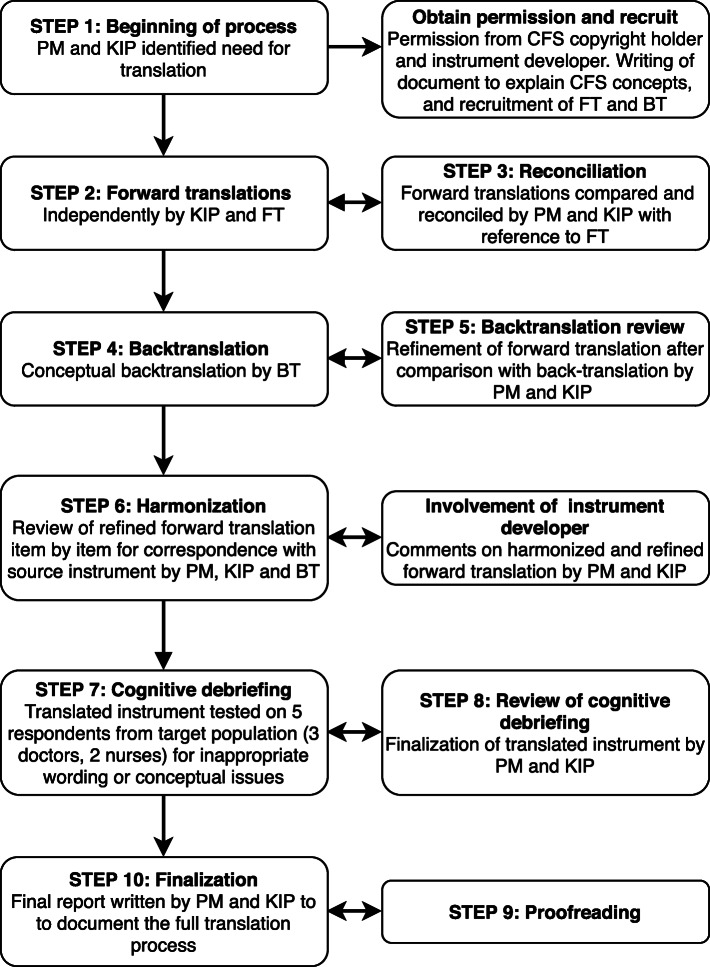
Flowchart of the Danish translation and cross-cultural adaptation of the Clinical Frailty Scale (CFS) following the 10-step ISPOR Principles of Good Practice for the Translation and Cross-Cultural Adaptation of Patient-Reported Outcomes [17]. Abbreviations: PM = project manager, KIP = key in-country person, FT = forward translator, BT = back translator

To ensure cultural and conceptual compliance with the source instrument [9, 11], we translated the CFS into the Danish language using the 10-step ISPOR Principles of Good Practice for the Translation and Cultural Adaptation of Patient-Reported Outcomes [17]. The Danish translation process is depicted in Fig. 2.

### Rating process

Health care professionals from the primary and secondary health care sectors were recruited to validate the CFS-DK and to support potential future use in a cross-sectoral context. Forty raters assessed 15 written clinical case vignettes using the CFS-DK. The raters were 10 community nurses, 10 general practitioners, and 20 hospital doctors (10 from internal medicine, 10 from intensive care) and were recruited as a convenience sample from the authors’ professional network.

Cases consisted of a short text and a picture of each case-patient and were selected to collectively represent all nine levels of the CFS. Cases provided essential information on 1) symptoms of diseases, 2) dependency on others, 3) cognitive function, and 4) physical condition. Each case was presented alongside a picture of the CFS-DK for reference (Fig. 1). Cases were built to imitate real-life patients by authors SKN (senior registrar in geriatric medicine) and KAR (consultant and professor in geriatric medicine). Prior to completing the questionnaire, raters were asked to view a five-minute video introducing frailty as a concept, and the CFS and its use in clinical practice, as well as raising awareness of pitfalls in using CFS, e.g. scoring patients with dementia. The raters then assessed each case according to the 9-point CFS-DK. The cases were presented in random order of severity but were rated in the same order by each rater. Finally, raters were asked to assess the relevance of the CFS for their own area of work and for cross-sectoral collaboration.

### Data collection

Study data were collected using an online questionnaire developed in REDCap (version: REDCap 9.1.15 -© 2020 Vanderbilt University) [18, 19], an electronic data capture tool hosted at Open Patient Exploratory Network (OPEN) at Odense University Hospital, the Region of Southern Denmark.

### Statistical analysis

Inter-rater reliability was assessed using the intraclass correlation coefficient (ICC) with 95% confidence intervals (CI) [20, 21] that was calculated i) for all 40 raters, and ii) within each professional group. The ICCs were considered poor (< 0.40), fair (0.40–0.59), good (0.60–0.75), or excellent (> 0.75) according to standard practice [22].

The sensitivity of the inter-rater reliability was examined using a random effect linear regression [23] with CFS-DK scores as outcome and with random effects included for case and rater, and with rater experience and professional group as covariates. The values from the community nurses were used as reference. In this way, we could assess the extent to which the inter-rater reliability was sensitive to the raters’ length of clinical experience and any unobserved variations in raters and cases. Other studies have used a graphical Bland-Altman approach [24], but this was less appropriate for the current study as our raters came from four different professional groups.

Statistical analysis was performed using SAS software (SAS Institute Inc., Cary, NC, USA). The statistical significance threshold for all tests was set to *P* < 0.05. Figures were made using “R” software (Version 3.6.1) [25] and the ggplot2 package [26].

## Results

### Translation process

In accordance with the ISPOR guidelines [17], the final report of the translation process is available in Additional file 1. The translation process is depicted in Fig. 2 and is summarized as follows:

Step 2–3: We observed high agreement in the meaning and wording of the two forward translations. Minor incongruities were mostly related to synonyms for particular words. Differences were discussed and resolved in a reconciliation meeting. Step 4–5: The back translation corresponded well to the reconciled forward translation. The few discrepancies were identified and discussed, leading to minor word changes in items 4, 5, 6, and 9.

Step 6: To ensure concordance between the harmonized translation and the source instrument, the original instrument developer was contacted for revision and feedback. This led to two minor changes in items 5 and 9. Step 7–8: To test for conceptual coherence, interpretation, and cultural relevance, the translated instrument was tested on five respondents from the target population (two general practitioners, one community nurse, and two geriatricians). Three respondents were women and two were men, and occupational experience ranged from 5 to 40 years. The five respondents were asked to score the same three cases, after which a cognitive debriefing was held with each respondent individually. This did not result in changes to the translation. Step 9–10: The final translation was proofread. The original source instrument and the Danish translation are presented in Fig. 1.

### Validation

All 40 raters assessed the 15 cases, yielding 600 observations with 40 replicate observations per case. Twenty-two of the 40 raters (55%) were women, and mean length of clinical experience in the four professional groups ranged from 14 to 17 years, with hospital doctors being the most experienced.

**Fig. 3:**
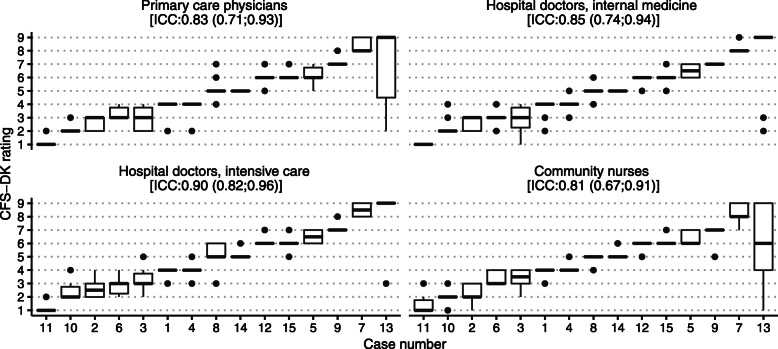
Boxplots of ratings on the Danish version of the Clinical Frailty Scale (CFS-DK) for 15 clinical case vignettes, by professional group and ordered after median scores. Thick horizontal lines represent median ratings; boxes show interquartile ranges; vertical lines represent minimum and maximum ratings; outliers are represented by circles. The intraclass correlation coefficient (ICC) for each professional group is presented with 95% CI

The overall inter-rater reliability for all 40 raters (based on individual assessments) was 0.85 (0.74; 0.93). As shown in Fig. 3, the inter-rater reliability was similar for the four professional groups, though highest for the hospital doctors specialized in intensive care (0.90, CI 0.82; 0.96). The ratings had narrow interquartile ranges and median ratings within one level for all but one case. The ratings for Case 13 (which described a case at level 9 with terminal illness but ‘not otherwise evidently frail’) had wide interquartile ranges for both primary care physicians and community nurses.

Table 1 shows the results of the random effect linear regression (with CFS-DK scores as outcome and with case and rater as unobserved random effects) aiming at examining the sensitivity of inter-rater reliability to rater experience. Although the hospital doctors specialized in intensive care came close to being significantly different (*p* = 0.05), the inter-rater reliability did not differ significantly between the professional groups or according to the length of the raters’ clinical experience (*p* = 0.96). The random effects components showed that variation between cases (4.40) was more important than variation between raters (0.01).

**Table 1:** Sensitivity analysis of cross-sectoral inter-rater reliability using multilevel regression modelling (with CFS-DK scores as outcome). Presented are linear regression coefficients (‘estimates’) with 95% confidence intervals (CI) for ratings according to professional group and length of clinical experience compared to the cross-sectoral regression coefficient. The random effects terms for raters and cases are also presented

Effect	Estimate	95% CI		***P*** value
Intercept	4.66	−16.05	17.15	< 0.01
Primary care physicians	0.06	−0.99	1.21	0.58
Hospital doctors, internal medicine	0.05	−0.75	0.97	0.66
Hospital doctors, intensive care	0.21	−3.66	3.87	0.05
Community nurses (reference)	0	.	.	.
Length of clinical experience	0.0002	−0.09	0.10	0.96
**Random effect variance terms**				
Raters	0.01			
Cases	4.40			
Residual	0.80			

All but one of the 40 raters considered the CFS-DK to be relevant for their own area of work, and all raters considered it to be relevant for cross-sectoral collaboration.

## Discussion

We successfully translated and validated the CFS into Danish (CFS-DK). The cross-sectoral validation found the CFS-DK to have excellent inter-rater reliability, both within each of the four health professional groups and between these groups. The health care professionals also considered the CFS-DK as being relevant to their area of work and to cross-sectoral collaboration.

These results suggest that the CFS-DK is a useful measure of frailty that can be applied meaningfully in both the primary and secondary health care sectors. Previous studies have provided evidence of good sensitivity and specificity of CFS [9] and its ability to predict a range of adverse health outcomes [12–16].

A valid and reliable measure of clinical frailty that can be used both within and across health care sectors has several advantages. It has the potential to improve collaborative efforts in the treatment, care, and rehabilitation of frail patients, who often need input from variety of health and social care providers. Reporting a standardized frailty measure by, for example, community nurses could enable primary care physicians to appreciate early signs of functional and physical deterioration among patients receiving home and social care. Hospital doctors and patients could benefit from a frailty assessment when determining treatment options and when planning hospital discharge and rehabilitation or end-of-life care. A standardized frailty measure might also assist community nurses to identify patients requiring extra follow-up after hospital treatment.

CFS is best suited as the entry point for intervention planning, as it is designed for cross-sectional assessments, rather than tracking trajectories [27]. The approach for cross-sectoral comparison of inter-rater reliability employed in this study could also be used for validation of trajectory tracking models.

In our study, the inter-rater reliability was very high (0.98). A recent validation study of the CFS in both English and French reported similar high inter-rater reliability of 0.87 (95%CI: 0.76–0.93) for native French doctors using the source CFS in English, and 0.76 (95%CI: 0.57–0.87) for native French nurses using the French translation of the CFS [24].

While these results correspond well with the observed inter-rater reliability for hospital doctors in this study, reliability evaluations should be interpreted with care as they depend on the assessment conditions [28], e.g. location, disturbances, rater characteristics and availability of information. Other psychometric properties of the CFS-DK, such as responsiveness and predictive validity, should also be tested in clinical conditions more similar to daily routine and on actual clinical cases, as recently demonstrated for the German version of the CFS [12]. This could be usefully done in combination with intervention studies, for example. Convergent validity of the CFS was tested during development against the Frailty Index [9] and could also be tested in different settings.

The estimates for inter-rater reliability in the current study would probably have been improved if we had separated out Case 13 that described level 9 on the CFS (terminally ill but otherwise no evidence of frailty). This level could have been further explained with a note at the start of the CFS tool, or specific training could have been provided on rating this particular level. An underlying assumption in the CFS is that life expectancy declines with increasing frailty from item 1 to item 9. However, functional dependency and frailty progress only from item 1 to item 8. At item 9, physical limitation is not apparent and, by definition, level 9 is “not otherwise evidently frail”. This likely confuses raters and can be observed in the wide distribution of ratings for Case 13. Another possible explanation is that Case 13 uses terms like “[lung cancer disseminated to multiple organs]” and “[declined palliative therapy]”, which require health care professionals recognizing the implied consequences (i.e. severe prognosis and short residual lifespan).

Calibration of the CFS scale is likely dependent on frailty incidence [29], and maybe in particular item 9 for different health care sectors. Use of the CFS within a hospital might be limited by a lack of discriminative ability for patients with severely affected functional level (i.e. in geriatric departments) as it was developed for community-dwelling adults aged 65 years and over, but no systematic reviews have yet been made of the prognostic performance of the CFS.

The responsibility for care in the frail population varies across European countries, and this has implications for the future use of the CFS. In the Scandinavian countries and the Netherlands, there is societal consensus for a welfare system in which the public sector is responsible for providing good and equal health and long-term social care [30–32]. However, demographic changes mean that responsibility for long-term care is slowly moving towards families and relatives, even in publicly financed health care systems [32]. Consulting family and relatives will thus probably be important when health care professionals use the CFS in the future.

### Strengths and limitations

This study used clinical case vignettes, and although the case vignettes were clinically appropriate and built to imitate real-life patients, they could also be regarded as hypothetical. Case vignettes provide the opportunity to let raters assess the exact same information, with the same structure and nearly identical setting at the same time point, allowing equal comparisons between professional groups. The cases were designed to encompass all the CFS levels. Ratings given to the 15 cases clearly reflect these different degrees of severity. Conversely, case vignettes introduce a risk of inflated inter-rater reliability and ICC as raters assess all cases at once, which does not reflect clinical practice.Another limitation to this study is the relatively small number of cases (*n* = 15). However, the use of case vignettes allowed a high number of observations of 600 (15 cases, 40 raters), a quadrupling compared to previous investigations of inter-rater reliability following translation of the CFS [24].

Finally, raters were recruited as a convenience sample, inherently risking inflated reliability measures and sensitivity analyses.

This study has several strengths. First, the translation process was completed using a rigorous procedure that followed the ISPOR guidelines. Poorly translated instruments threaten the validity of the data, and quality is dependent on methodology [17]. Second, the validation included key actors most often involved in significant transitions between the primary and secondary health care sectors, and the health care professionals were experienced in their clinical fields. Third, raters were informed that inter-rater reliability would be compared between professional groups but that individual rater performance would not be assessed. We expect this to have reduced the risk of a Hawthorne effect (i.e. awareness of being observed).

## Conclusion

The Clinical Frailty Scale was translated and culturally adapted into Danish following a careful and well-established standard process. The inter-rater reliability was high in all four groups of health care professionals involved in cross-sectoral collaborations. However, the use of case vignettes may reduce the generalizability of the reliability findings to real-life settings. The CFS has the potential to serve as a common reference tool when treating and rehabilitating older patients.

## Supplementary Information

**Additional file 1.**

## Data Availability

The datasets used and/or analysed during the current study are available from the corresponding author on reasonable request.

## Abbreviations

BT: Back translator
CFS: Clinical Frailty Scale
CFS-DK: Clinical Frailty Scale - Danish version
CI: Confidence interval
FT: Forward translator
IADL: Instrumental Activities of Daily Life
ICC: Intraclass correlation coefficient
IQR: Interquartile range
KIP: Key in-country person
PM: Project manager
