# Supplementary material for: Cross-sectoral inter-rater reliability of the clinical frailty scale – a Danish translation and validation study
Source: BMC Geriatr. 2020 Nov 3;20:443. doi: 10.1186/s12877-020-01850-y (PMC7640648; doi:10.1186/s12877-020-01850-y)
Supplement: Supplementary file 1 — Additional file 1. [file 12877_2020_1850_MOESM1_ESM.pdf]

Final report

Translation of Clinical Frailty Scale into the Danish language

Introduction

Demographic changes are increasingly challenging health care systems in most European countries. As humans age, the risk of morbidity and functional decline increases, followed by needs for primary and secondary health care services such as home care and hospital treatment. These changes are strongly associated with frailty, which represents a state of extreme vulnerability where minimal stress can cause functional impairment.

A unified and standardized frailty evaluation is needed to support treatment, care and rehabilitation of older patients across the primary and secondary health care sectors. Therefore, we aim to translate the Clinical Frailty Scale (CFS) into Danish using a systematic approach, with the aim of enabling its further use in research and cross-sectoral implementation in a Danish context.

The translation of the CFS were performed using the 10-step ISPOR Principles of Good Practice for the Translation and Cultural Adaptation Process for Patient-Reported Outcomes Measures (Wild et al. 2005).

Study group

Project manager: Anders Fournaise (AF), MSc Public Health, Industrial PhD-student, Region of Southern Denmark
Key In-country person: Søren Kabell Nissen (SKN), MD, PhD-student, Hospital of South West Jutland
Forward translation: Jessica Joan Williams (JJW), CNS, MSc in Public Health, University of Southern Denmark
Back translation: Claire Gudex (CG), MD, PhD, University of Southern Denmark
Proof-reading: Christina Boesen Kristensen (CBK), MA in Danish, Region of Southern Denmark
In-country consultant: Karen Andersen-Ranberg (KAR), Professor, MD, PhD, Odense University Hospital

Step 1: Preparation

Permission was obtained from the source instrument developer (appendix 1 and 2) by AF and SKN. Arrangements were made allowing for feedback from the source instrument developer at a later stage in the process.
The concept of frailty was investigated through a scoping review of relevant literature. The original development study was also investigated in detail (Ref: Rockwood 2005, CMAJ). AF and SKN then recruited researchers with key competences and informed them about the process to

Contact information: Anders Fournaise – anders.fournaise@rsyd.dk / Søren Kabell Nissen – sknissen@health.sdu.dk

secure a coordinated and complete translations process (see members of the study group above).

Step 2: Forward Translation

Two forward translations (appendix 3.1 and 3.2) were done by SKN (Danish native speaker, proficient in English, residing in DK) and JJW (English native speaker, proficient in Danish, residing in DK) independently after background information and explanations of concepts was provided by AF.

Step 3: Reconciliation

Reconciliation of the forward translations into a reconciled forward translation was performed by AF, SKN and KAR. The reconciliation was carried out as a discussion using video communication due to the SARS-CoV-2 pandemic.
Overall, the two forward translations had high agreement. The reconciliation for each item is described in detail in appendix 4.

Step 4: Back-translation

The back translation (appendix 5) was performed by CG, who is associate professor in health services research and a medical writer experienced in translation guidelines and instrument validation. CG was asked for a conceptual back translation by AF.

Step 5: Back-translation review

To ensure conceptual equivalence of the translation, AF and SKN reviewed the back translation against the original Clinical Frailty Score (source instrument). The back translation was sent to the developer of source instrument for revision and feedback. No major discrepancies were identified, and the reconciled translation was only slightly refined.
See appendix 6 for a detailed review of the back translation and appendix 7 for the feedback from the designer of source instrument.

Step 6: Harmonization

The minor changes proposed in the back-translation review were discussed in a harmonization meeting between AF, SKN and CG. Close attention was paid to the correspondence of each back-translated item and the source instrument. Appendix 8 provides an overview of the changes made to the reconciled translation. The product of steps 3 and 5, the harmonized version, is presented in appendix 8.1.

Step 7: Cognitive debriefing

A cognitive debriefing was completed to explore alternative wording and to check for understandability, interpretation and cultural relevance of the translation.

To test the harmonized Danish translation of the CFS, we asked five health personnel (a senior consultant from a hospital geriatric department, a hospital registrar, a general practitioner, a hospital nurse, and a community nurse) to complete three cases and report on their use of the CFS-DK. The assessment was completed using an online questionnaire built into REDCap (version: REDCap 9.1.15 - © 2020 Vanderbilt University). Redcap is an electronic data capture

Contact information: Anders Fournaise – anders.fournaise@rsyd.dk / Søren Kabell Nissen – sknissen@health.sdu.dk

tool hosted at Open Patient data Exploratorive Network (OPEN) at Odense University Hospital, the Region of Southern Denmark.

AF and SKN completed a debriefing as either a video or telephone meeting with each of the five assessors to gather their feedback.

Step 8: Review of cognitive debriefing results and finalization

No changes were made to the translation based on the review of the cognitive debriefing. The review did lead to a few changes of the introduction materiale explaining the CFS concept. This included minor changes to a short video presented to the raters before using the online questionnaire.

Step 9: Proofreading

The final translation (appendix 9) was proofread by CBK who holds a Master of Sciences in Danish. This resulted in minor corrections of grammatical errors.

Step 10: Conclusions

To conclude the translation process, SKN and AF drafted this final report, which was commented and approved by the study group. To strengthen the basis for implementation of the CFS in the cross-sectoral collaboration the study group has begun a validation of the CFS_DK. The translation process and results of the validation will be disseminated in an article, which will be submitted to a peer-reviewed journal.

Acknowledgements

The study group wish to acknowledge Jens Vestergaard, Katja Thomsen, Lars Matzen, Line Emilie Laugesen and Susan Feldborg for participating in the cognitive debriefing and later validation of the CFS-DK.

Further we wish to acknowledge the following for the contribution to the validation of the CFS_DK: Anders Lassen, Lisbeth Rossel, Christel Kronborg, Elisabeth Schmidt, Anna Nilsson, Helle Nilsson, Ditte De Churruca, Pia Therkildsen, Emil Hanson, Tone Sollie, Dorthe Schach, Mette Kock, Sanne Juul Nikolajsen, Pernille Seekjær, Rikke Hansen, Tina Ellegaard, Ditte Bødker, Dorrit Borgnæs, Jens-Ulrik Rosholm, Søren Kasch, Stine Hanson, Peter Frederiksen, Marianne Fløjstrup, Thomas Veedfald, Martin Rahbek, Anne Nissen, Christian Svendsen Juhl, Cheme Andersen, Bjarne Skjødt Hjaltelin, Finn Østergaard Bærentzen, Jacob Vad Jensen, Jakob Hessel Andersen, Kim Zillo Rokamp, Lars Nebrich, Valerij Khridin & Lone Musaeus Poulsen.

Finally, the authors also acknowledge David Hass from OPEN, Open Patient data Explorative Network, Odense University Hospital, Region of Southern Denmark for assistance in data management.

Contact information: Anders Fournaise – anders.fournaise@rsyd.dk / Søren Kabell Nissen – sknissen@health.sdu.dk

Appendix 1 - Source instrument

Clinical Frailty Scale*

1 **Very Fit** – People who are robust, active, energetic and motivated. These people commonly exercise regularly. They are among the fittest for their age.

2 **Well** – People who have **no active disease symptoms** but are less fit than category 1. Often, they exercise or are very **active occasionally**, e.g. seasonally.

3 **Managing Well** – People whose **medical problems are well controlled,** but are **not regularly active** beyond routine walking.

4 **Vulnerable** – While **not dependent** on others for daily help, often **symptoms limit activities.** A common complaint is being "slowed up", and/or being tired during the day.

5 **Mildly Frail** – These people often have **more evident slowing,** and need help in **high order IADLs** (finances, transportation, heavy housework, medica-tions). Typically, mild frailty progressively impairs shopping and walking outside alone, meal preparation and housework.

6 **Moderately Frail** – People need help with **all outside activities** and with **keeping house**. Inside, they often have problems with stairs and need **help with bathing** and might need minimal assistance (cuing, standby) with dressing.

7 **Severely Frail** – **Completely dependent for personal care**, from whatever cause (physical or cognitive). Even so, they seem stable and not at high risk of dying (within ~ 6 months).

8 **Very Severely Frail** – Completely dependent, approaching the end of life. Typically, they could not recover even from a minor illness.

9. Terminally Ill - Approaching the end of life. This category applies to people with **a life expectancy <6 months**, who are **not otherwise evidently frail.**

Scoring frailty in people with dementia

The degree of frailty corresponds to the degree of dementia. Common **symptoms in mild dementia** include forgetting the details of a recent event, though still remembering the event itself, repeating the same question/story and social withdrawal.

In **moderate dementia**, recent memory is very impaired, even though they seemingly can remember their past life events well. They can do personal care with prompting.

In **severe dementia**, they cannot do personal care without help.

* 1. Canadian Study on Health & Aging, Revised 2008.
2. K. Rockwood et al. A global clinical measure of fitness and frailty in elderly people. CMAJ 2005;173:489-495.

© 2009. Version 1.2_EN. All rights reserved. Geriatric Medicine Research, Dalhousie University, Halifax, Canada. Permission granted to copy for research and educational purposes only.

DALHOUSIE UNIVERSITY *Inspiring Minds*

Contact information: Anders Fournaise – anders.fournaise@rsyd.dk / Søren Kabell Nissen – sknissen@health.sdu.dk

Appendix 2 - Copyright permission

DALHOUSIE UNIVERSITY

FOR OFFICE USE ONLY
PF#: 20200316 - 05

PERMISSION TO USE THE CLINICAL FRAILTY SCALE (CFS)©

The undersigned is granted permission to use, reproduce and distribute the Clinical Frailty Scale (CFS)© in the format attached[1] for educational purposes and for non-commercially funded research and/or quality assurance projects. The CFS© must be administered free of charge to patients and/or study participants.

A formal Licensing Agreement is required for research funded by any commercial entity or pharma and, in some cases, for use in routine clinical care.

The copyright holder reserves the right to prospectively follow-up at any time to determine whether use of the CFS© meets the conditions described above.

Reselling of the CFS© or other commercial development without a license agreement is prohibited by copyright.

The undersigned, their delegates and affiliated organization(s) agree that they will not claim ownership rights to the CFS©, or any derivative, including translations, compilation, sequel or series. Nothing in this Agreement shall give the undersigned any right, title, or interest in the CFS© other than the right to use in accordance with this Agreement. The CFS© will not be modified unless explicitly given permission to do so.

**USER INFORMATION:**	
Full Name:	Soren Kabell Nissen
Position/Title:	MD, PhD student
Institution/Organization:	Institute for Health Research, University of Southern Denmark
Mailing Address:	Finsensgade 35, 6700 Esbjerg, Denmark
Telephone:	+45 21 80 38 17
Email:	SKNissen@health.sdu.dk
Type of organization:	☐ For-profit ☒ Not-for-profit ☐ Other, *please specify:*

INTENDED USE (Select all that apply):

☐ Reprint — *Provide publication details:*

☒ Research study / clinical trial — *Describe use in study:* 1. Perform translation to Danish according to the standards suggested by the ISPOR Task Force for Translation and Cultural Adaptation. 2. Validate in Danish primary care setting as part of a randomized controlled clinical trial.
Expected duration of study: Start date 04.2020 End date 05.2021

☐ Routine clinical care
Will the CFS© be incorporated into an electronic medical health record (EMR)? ☐ Y ☐ N
***If yes**, please identify whether an EMR company is involved (e.g., Epic, Meditech):*

☐ Other — *Specify:*

Are you planning to translate the CFS©? ☒ Y ☐ N ***If yes**, specify language(s):* Danish
We request editable (e.g. MSWord) copies of all translations. We do not independently verify or validate translations.

Are you planning any commercial development that would incorporate the CFS©?
☐ Y ☒ N ***If yes**, please specify:*

By your signature below, you attest that you understand the conditions under which permission is granted.

Signature:	[signature]
Date:	03.11.2020

Send completed and signed form by post/fax or email to:	Kenneth Rockwood, MD<br>1421-5955 Veterans' Memorial Lane<br>Halifax, NS B3H 2E1 Canada<br>Fax: 1-902-473-1050 | Email: gmru@dal.ca

Approved by: [signature] Kenneth Rockwood, MD (or delegate) Date: 2020/03/16

[1] A copy of the CFS will be emailed to the user upon review and approval of this permission form. Valid only when signed by all parties.

CFS© Permission For Use Form V6 2019Sept8

Contact information: Anders Fournaise – anders.fournaise@rsyd.dk / Søren Kabell Nissen – sknissen@health.sdu.dk

Appendix 3.1 - Forward-translation 1

Performed by JJW

1. Meget god form – Folk, der er robuste, aktive, energiske, og motiverede. Disse folk træner ofte regelmæssigt. De er blandt dem i bedst form for deres alder.

2. Godt – Folk, der ikke har symptomer på sygdom i udbryd, men er i mindre god form end folk i kategori 1. Disse folk træner ofte, eller er meget aktive regelmæssigt, f.eks. på bestemte årstider.

3. Klarer sig godt – folk, hvis sygdomsproblemer er velkontrollerede, men som ikke er aktive regelmæssigt udover rutinemæssige gåtur.

4. Sårbar – Imens ikke afhængigt af andre i forbindelse med dagligdagsaktiviteter, symptomer ofte hæmmer aktiviteter. En almindelig klage er at være 'lidt langsommere' og/eller at være træt i løbet af dagen.

5. Mildt skrøbelig – Disse folk er mere tydeligt langsomme og har brug for hjælp, hvad der angår 'high order IADLs' (finanser, transport, hovedrengøring, medicin). Mildt skrøbelighed ofte gradvist forhindrer det at gå ud og handle, udendørs gåtur alene, madlavning og husarbejde.

6. Moderat skrøbelig – Folk, der har brug for hjælp med alle udendørs aktiviteter og husarbejde. Indendørs har de ofte problemer med trapper og har brug for hjælp til at komme i bad og eventuelt også minimal assistance (guidning, støtte) til at tage tøj på.

7. Alvorligt skrøbelig – fuldstændig afhængig, hvad der angår personlig pleje, uanset grunden herfor (fysisk eller kognitivt). Til gengæld forekommer de stabile og ikke i høj risiko for at dø (inden for ca. 6 måneder).

8. Meget alvorligt skrøbelig - fuldstændig afhængig og ved at nærme sig livets ende. Typisk vil de ikke kunne komme sig efter selv en mild sygdom.

9. Terminal – er ved at nærme sig livets ende. Denne kategori gælder for folk med en forventet levealder på <6 måneder, og som ellers ikke er tydeligt skrøbelige.

AFSNIT OM DEMENS:

At score skrøbelighed(en) hos personer med demens

Graden af skrøbelighed(en) svarer til graden af demens.

Almindelige symptomer på mild demens inkluderer det at glemme detaljerne på en nylig begivenhed, imens man stadig kan huske selve begivenheden, det at gentage det samme spørgsmål/fortælling, og social isolation.

I moderat demens, den nylige hukommelse er meget forringet, selv om man synes at kunne huske gamle minder og begivenheder godt. Man kan udføre personlig pleje ved hjælpe af guidning.

I alvorlig demens kan man ikke udføre personlig pleje uden hjælp.

Contact information: Anders Fournaise – anders.fournaise@rsyd.dk / Søren Kabell Nissen – sknissen@health.sdu.dk

Appendix 3.2 - Forward-translation 2

Performed by SKN

1: **Meget god form** – Mennesker der er robuste, aktive, energiske og motiverede. Disse mennesker motionerer regelmæssigt. De er blandt dem der er mest sunde i forhold til alderen.

2: **Velbefindende** – Mennesker **uden aktive tegn på sygdom**, men ikke er i så god form som kategori 1. De motionerer eller er meget **aktive en gang imellem**, eksempelvis afhængig af sæson.

3: **Klarer sig godt** – Mennesker med **vel kontrollerede sygdomme**, men **ikke regelmæssigt aktive** udover gåture.

4: **Sårbare** – Mennesker der **ikke er afhængige** af andre til daglige gøremål, men ofte har **symptomer der begrænser aktiviteterne**. En hyppig klage er at føle sig "langsom" eller træt i løbet af dagen.

5: **Mildt skrøbelige** – Mennesker der ofte er **mere tydeligt langsomme**, og har **behov for hjælp til kompekse IADL'er** (økonomi, transport, tungt husarbejde, mediciner). Typisk vil mild skrøbelighed hæmme indkøb, gåture alene udenfor, madlavning og husarbejde.

6: **Moderat skrøbelige** – Mennesker med behov for hjælp til **alle udendørs aktiviteter** og med at **holde hus**. Indenfor har de problemer med trapper og har behov for **hjælp til at gå i bad** og har måske behov for minimal (stikord, standby) hjælp til påklædning.

7: **Svært skrøbelige – Fuldstændig afhængige afhjælp til egenomsorg**, uanset årsag (fysisk eller kognitiv). Alligevel virker de stabile og ikke i høj risiko for at dø (indenfor ~ 6 måneder).

8: **Meget svært skrøbelige** – Fuldstændig afhængige og nærmer sig livets afslutning. Typisk vil de ikke engang kunne komme sig efter let sygdom.

9: **Terminalt syge** – Nærmer sig livets afslutning. Denne kategori gælder mennesker med en **forventet levetid på < 6 måneder**, og som **ikke fremstår tydeligt skrøbelige i øvrigt**.

Bedømmelse af skrøbelighed ved demens

Graden af skrøbelighed svarer til graden af demenssymptomer.

Typiske **symptomer ved mild demens** er at glemme detaljer om en nylig begivenhed, selvom man kan huske selve begivenheden, at gentage de samme spørgsmål/historie og social tilbagetrækning.

Ved **moderat demens** er hukommelsen for nylige begivenheder svært nedsat, selvom de tilsyneladende kan huske tidligere livsbegivenheder tydeligt.

De kan udføre personlig hygiejne uden at spørge om hjælp.

Ved **svær demens** kan de ikke udføre personlig hygiejne uden hjælp.

Contact information: Anders Fournaise – anders.fournaise@rsyd.dk / Søren Kabell Nissen – sknissen@health.sdu.dk

Appendix 4 - Reconciliation

Performed by SKN, KAR and AF

Item 1 decisions:

- “Mennesker” instead of “Personer” was chosen
- “Motionerer” instead of “Træner” was chosen, as the latter implies a framed setting like attending a gym-class or similar
- “Bedst form” instead of “Bedst helbred” as this item related to function rather than diseases.

Item 2 decisions:

- “Symptomer” instead of “Tegn” was chosen as “tegn” implies indications of undiagnosed disease rather than manifest symptoms
- “En gang imellem” instead of “regelmæssigt”, as the latter implies a more frequent occurrence than occasionally.

Item 3 decisions:

- Added “rutinemæssige” to this item as proposed by JJW.

Item 4 decisions:

- “Almindelig” instead of “Hyppig” as the latter implies a very frequent occurrence rather than simply frequent

Item 5 decisions:

- “IADL” was deselected for “daglige gøremål” as IADL is a technical term and abbreviation not necessarily familiar to alle health care professionals for whom this instrument has its intended use.

Item 6 decisions:

- “Indendørs” instead of “indenfor” as it is considered easier to conceptualise for raters
- “Trappegang” instead of “trappe” as the latter implies troubles with stairs other than simply troubles walking up stairs, risking loss of relevance in relation to physical functioning
- Added “nogle gange” to underline the intent of the source instrument

Item 7 decisions:

- Near complete agreement, no reconciliation needed

Item 8 decisions:

- Removed “kunne” as this word is not considered necessary for understanding

Item 9 decisions:

- Starting with “Mennesker som ikke fremstår tydeligt skrøbelige i øvrigt” used in the final sentence by JJW as in Danish new complex units of information are rarely used at the end of sentences.

Item on dementia, decisions:

- Made the second sentence less complex. Simply stating that people with dementia are always frail “Mennesker med demens er skrøbelige”

Contact information: Anders Fournaise – anders.fournaise@rsyd.dk / Søren Kabell Nissen – sknissen@health.sdu.dk

Appendix 4.1 - Reconciled version

1: **Meget god form** – Mennesker der er robuste, aktive, energiske og motiverede. Disse mennesker motionerer regelmæssigt. De er blandt dem i bedst form for deres alder.

2: **Velbefindende** – Mennesker **uden aktive symptomer på sygdom**, men i mindre god form end kategori 1. De motionerer ofte eller er meget **aktive en gang imellem**, f.eks. bestemte årstider.

3: **Klarer sig godt** – Mennesker med **velkontrollerede sygdomsproblemer**, men som **ikke er regelmæssigt aktive** udover rutinemæssige gåture.

4: **Sårbar** – Mennesker der **ikke er afhængige** af andre til daglige gøremål, men ofte har **symptomer der begrænser aktiviteterne**. En almindelig klage er at føle sig "langsom" eller træt i løbet af dagen.

5: **Mildt skrøbelig –** Mennesker der er **mere tydeligt langsomme,** og har **behov for hjælp til komplekse daglige gøremål** (økonomi, transport, hovedrengøring, medicin). Typisk vil mild skrøbelighed hæmme indkøb, gåture alene udenfor, madlavning og husarbejde.

6: **Moderat skrøbelig** – Mennesker med behov for hjælp til **alle udendørs aktiviteter** og med at **holde hus**. Indendørs har de ofte problemer med trappegang, **hjælp til at gå i bad** og nogle gange minimal hjælp til påklædning (stikord, let støtte).

7: **Svært skrøbelig** – **Fuldstændig afhængige af hjælp til egenomsorg**, uanset årsag (fysisk eller kognitiv). Alligevel virker de stabile og ikke i høj risiko for at dø (indenfor ~ 6 måneder).

8: **Meget svært skrøbelig** – Fuldstændig afhængige og nærmer sig livets afslutning. Typisk vil de ikke engang komme sig efter let sygdom.

9: **Terminalt syg** – Mennesker som ikke fremstår tydeligt skrøbelige i øvrigt. Denne kategori gælder mennesker med en **forventet levetid på < 6 måneder**.

Bedømmelse af skrøbelighed hos mennesker med demens:

Mennesker med demens er skrøbelige, graden af demens svarer til graden af skrøbelighed

Typiske **symptomer ved mild demens** er at glemme detaljer om nylige begivenheder, selvom man kan huske selve begivenheden at gentage de samme spørgsmål/historier og social tilbagetrækning.

Ved **moderat demens** er hukommelsen for nylige begivenheder svært nedsat, selvom de tilsyneladende kan huske gamle minder tydeligt. De kan udføre personlig pleje med vejledning.

Ved **svær demens** kan de ikke udføre personlig pleje uden hjælp.

Contact information: Anders Fournaise – anders.fournaise@rsyd.dk / Søren Kabell Nissen – sknissen@health.sdu.dk

Appendix 5 - Back-translation

Performed by CG

1: Very good shape (Very fit) - People who are robust, active, energetic and motivated. These people exercise regularly. They are among those in the best shape (among the fittest) for their age.

2: In good shape (Feeling well) - People **without active symptoms of illness**, but in less good shape (less fit) than category 1. They exercise often or are very active once in a while, e.g. at certain times of the year.

3: Manage (cope) well - People with **well-controlled medical conditions** but are not regularly active apart from routine walks.

4: Vulnerable - People who are **not dependent** on others for daily activities but often have symptoms that restrict their activities. A common complaint is feeling "slow" or tired during the day.

5: Mildly fragile - People who are **more clearly slow** and **need help with complex daily activities** (finances, transport, heavy housework, medicine). Mild fragility typically limits shopping, walking alone outside, cooking and housework.

6: Moderately fragile - People who need help with **all outdoor activities** and with housekeeping. Indoors, they often have trouble with stairs, **need help with bathing** and sometimes minimal help with dressing (prompts, helping hand/slight support).

7: Severely fragile - **Fully dependent on others for personal care**, for whatever reason (physical or cognitive). However, they appear stable and not at high risk of dying (within ~ 6 months).

8: Very severely fragile – Completely dependent and nearing the end of life. They will typically not recover from even slight illness.

9: Terminally ill - People who otherwise do not appear fragile. This category applies to people with a **life expectancy of <6 months**.

Assessing the fragility of people with dementia:
People with dementia are fragile, and the degree of dementia corresponds to the degree of fragility.

Typical **symptoms of mild dementia** are forgetting the details of recent events although remembering the event itself, repeating the same questions/stories and social withdrawal.

People with **moderate dementia** have severely impaired memory of recent events even though they seem to remember old memories clearly. They can perform personal care with guidance.

People with **severe dementia** cannot perform personal care without help.

Contact information: Anders Fournaise – anders.fournaise@rsyd.dk / Søren Kabell Nissen – sknissen@health.sdu.dk

Appendix 6 - Back-translation review

Performed by AF and SKN

Item 1

- No significant conceptual discrepancies

Item 2

- No significant conceptual discrepancies, not necessary to refine translation

Item 3

- No significant conceptual discrepancies, not necessary to refine translation

Item 4

- Minor conceptual discrepancy in regards to consideration of cases where subjects feel both "slow" or tired during the day.

Item 5

- "Fragile" has the same conceptual meaning in the danish language as "Frail" in the source instrument. Hence we do not consider this item to hold significant discrepancies.

Item 6

- Adding "kan nogle gange have brug for minimal"

Item 7

- No significant conceptual discrepancies, not necessary to refine translation

Item 8

- No significant conceptual discrepancies, not necessary to refine translation

Item 9

- The sentence-construction has been reversed, but specifically caters for cultural adaption

Item on dementia

- No significant conceptual discrepancies, not necessary to refine translation

Contact information: Anders Fournaise – anders.fournaise@rsyd.dk / Søren Kabell Nissen – sknissen@health.sdu.dk

Appendix 7 - Feedback from the designer of source instrument

Smith, Jeremy [Jeremy.Smith@nshealth.ca]
Handlinger
Til: Søren Kabell Nissen [sknissen@health.sdu.dk]
Cc: gmru@dal.ca; Anders Fournaise

23. april 2020 19:31

Hi Søren and Anders,

Here are some comments regarding the differences between our original English translation and the back-translation:

1 Very Fit - Differences are fine.

2 Well - Differences are fine.

3 Managing well - Good.

4 Vulnerable - Good.

5 Mildly Frail - Difference: Progressively impairs (original) vs. limits. From Dr. Olga Theou, "I think progressively adds something to this, is it able to be added?"

6 Moderately frail - Good.

7 Severely Frail - Good.

8 Very Severely Frail - Good.

9 Terminally Ill - Dr. Olga Theou, "Why not approaching the end of life added? Unless it is not culturally appropriate. Then it is fine. Even so, not otherwise evidently frail is important for this definition and should be added."

Cheers and let me know if you have any other questions,

Jeremy Smith
Research Assistant

Contact information: Anders Fournaise – anders.fournaise@rsyd.dk / Søren Kabell Nissen – sknissen@health.sdu.dk

Appendix 8 - Harmonization

Participants: AF, SKN and CG
Literal translations are placed in brackets [example] for explanation of context.
Minor adjustments from plural to singular in items 6-9

Item 1

- "Typisk" was added to enhance correspondence to the source instrument where the word "Commonly" is found

Item 2

- No changes added

Item 3

- No changes added

Item 4

- Minor conceptual discrepancy to source instrument in regards to consideration of cases where subjects feel both "slowed up" and/or tired during the day. In backwards translation "and/or" is lost, but the group believes the translation still retains correspondence with the source instrument without "og/eller" ["and/or"].

Item 5

- The back translator prefers the term "frail" rather than "fragile", used in the back translation, noting that the latter most often relates to things, rather than people. This does not affect the Danish translation as "fragile" has the same conceptual meaning in Danish as "frail" in the source instrument.
- The term "progressively" in the source instrument is missing, adding "i stigende grad" [increasingly] to the harmonized version, "progressively" is not considered commonly understood for Danish natives.
- It was decided that "daglige gøremål" corresponds well to "IADL" from source instrument.
- "IADL" is added to the parenthesis with examples that correspond well to source instrument.

Item 6

- The term "standby" in source instrument does not correspond well, adding "ved behov" [if needed] inside parenthesis to "let støtte" [light support] as standby has no directly equivalent Danish term.
- Adding "kan" [can] to final sentence.

Item 7

- "~" changed to "ca.", as the tilde-sign is very rarely used in Danish texts outside of mathematics or programming.

Item 8

Contact information: Anders Fournaise – anders.fournaise@rsyd.dk / Søren Kabell Nissen – sknissen@health.sdu.dk

- No changes added

Item 9

- The sentence construction is reversed, but this specifically caters for cultural adaption.
- “<” is changed to “mindre”, as this sign has proven difficult in the daily use of previously translated instruments.

Item on dementia

- Adding “(scorer altid minimum 5)” [scores always minimum 5] to the sentence “Mennesker med demens er skrøbelige (scorer altid minimum 5)”. The logical implication that CFS will always be 5 or above for people with dementia needed to be concretized, this might reduce misinterpretation and increase correspondence with the source tool.

Contact information: Anders Fournaise – anders.fournaise@rsyd.dk / Søren Kabell Nissen – sknissen@health.sdu.dk

Appendix 8.1 - Harmonized version

1: **Meget god form** – Mennesker der er robuste, aktive, energiske og motiverede. Typisk motionerer disse mennesker regelmæssigt. De er blandt dem i bedst form for deres alder.

2: **Velbefindende** – Mennesker **uden aktive symptomer på sygdom**, men i mindre god form end kategori 1. De motionerer ofte eller er meget **aktive en gang imellem**, f.eks. på bestemte årstider.

3: **Klarer sig godt** – Mennesker med **velkontrollerede sygdomsproblemer**, men som **ikke er regelmæssigt aktive** udover rutinemæssige gåture.

4: **Sårbar** – Mennesker der **ikke er afhængige** af andre til daglige gøremål, men som ofte har **symptomer, der begrænser aktiviteterne**. En almindelig klage er at føle sig "langsom" eller træt i løbet af dagen.

5: **Mildt skrøbelig** – Mennesker der er **mere tydeligt langsomme**, og som har **behov for hjælp til komplekse daglige gøremål** (IADL - økonomi, transport, hovedrengøring, medicin). Typisk vil mild skrøbelighed i stigende grad hæmme indkøb, gåture alene udenfor, madlavning og husarbejde.

6: **Moderat skrøbelig** – Mennesker med behov for hjælp til **alle udendørs aktiviteter** og med at **holde hus**. Indendørs har de ofte problemer med trappegang, **hjælp til at gå i bad** og kan nogle gange have brug for minimal hjælp til påklædning (stikord, let støtte ved behov).

7: **Svært skrøbelig** – **Fuldstændig afhængige af hjælp til egenomsorg**, uanset årsag (fysisk eller kognitiv). Alligevel virker de stabile og ikke i høj risiko for at dø (indenfor ca. 6 måneder).

8: **Meget svært skrøbelig** – Fuldstændig afhængige og nærmer sig livets afslutning. Typisk vil de ikke engang komme sig efter let sygdom.

9: **Terminalt syg** – Mennesker som nærmer sig livets afslutning, men ikke fremstår tydeligt skrøbelige i øvrigt. Denne kategori gælder mennesker med en **forventet levetid på mindre 6 måneder**.

Bedømmelse af skrøbelighed hos mennesker med demens:

Mennesker med demens er skrøbelige (scorer altid minimum 5) og graden af skrøbelighed svarer til graden af demens.

Typiske **symptomer ved mild demens** er at glemme detaljer om en nylig begivenhed, selvom man kan huske selve begivenheden, og at gentage det samme spørgsmål/historie og social tilbagetrækning.

Ved **moderat demens** er hukommelsen for nylige begivenheder svært nedsat, selvom man tilsyneladende kan huske gamle minder tydeligt. Man kan udføre personlig pleje med vejledning.

Ved **svær demens** kan man ikke udføre personlig pleje uden hjælp.

Contact information: Anders Fournaise – anders.fournaise@rsyd.dk / Søren Kabell Nissen – sknissen@health.sdu.dk

Appendix 9 - Final version

Clinical Frailty Scale*

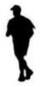

1 Meget god form – Mennesker der er robuste, aktive, energiske og motiverede. Typisk motionerer disse mennesker regelmæssigt. De er blandt dem i bedst form for deres alder.

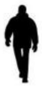

2 Velbefindende – Mennesker **uden aktive symptomer på sygdom**, men i mindre god form end kategori 1. De motionerer ofte eller er meget **aktive en gang imellem**, f.eks. på bestemte årstider.

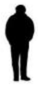

3 Klarer sig godt – Mennesker med **velkontrollerede sygdomsproblemer**, men som **ikke er regelmæssigt aktive** udover rutinemæssige gåture.

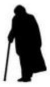

4 Sårbar – Mennesker der **ikke er afhængige** af andre til daglige gøremål, men som ofte har **symptomer, der begrænser aktiviteterne**. En almindelig klage er at føle sig "langsom" eller træt i løbet af dagen.

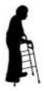

5 Mildt skrøbelig – Mennesker der er **mere tydeligt langsomme**, og som har **behov for hjælp til komplekse daglige gøremål** (IADL - økonomi, transport, hovedrengøring, medicin). Typisk vil mild skrøbelighed i stigende grad hæmme indkøb, gåture alene udenfor, madlavning og husarbejde.

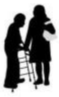

6 Moderat skrøbelig – Mennesker med behov for hjælp til **alle udendørs aktiviteter** og med at **holde hus**. Indendørs har de ofte problemer med trappegang, **hjælp til at gå i bad** og kan nogle gange have brug for minimal hjælp til påklædning (stikord, let støtte ved behov).

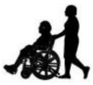

7 Svært skrøbelig – Fuldstændig afhængige af hjælp til egenomsorg, uanset årsag (fysisk eller kognitiv). Alligevel virker de stabile og ikke i høj risiko for at dø (indenfor ca. 6 måneder).

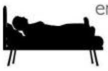

8 Meget svært skrøbelig – Fuldstændig afhængige og nærmer sig livets afslutning. Typisk vil de ikke engang komme sig efter let sygdom.

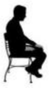

9 Terminalt syg – Mennesker som nærmer sig livets afslutning, men ikke fremstår tydeligt skrøbelige i øvrigt. Denne kategori gælder mennesker med en **forventet levetid på mindre end 6 måneder.**

Bedømmelse af skrøbelighed hos mennesker med demens

Mennesker med demens er skrøbelige (scorer altid minimum 5) og graden af skrøbelighed svarer til graden af demens.
Typiske **symptomer ved mild demens** er at glemme detaljer om en nylig begivenhed, selvom man kan huske selve begivenheden, og at gentage det samme spørgsmål/historie og social tilbagetrækning.

Ved **moderat demens** er hukommelsen for nylige begivenheder svært nedsat, selvom man tilsyneladende kan huske gamle minder tydeligt. Man kan udføre personlig pleje med vejledning.

Ved **svær demens** kan man ikke udføre personlig pleje uden hjælp.

* 1. Canadian Study on Health & Aging, Revised 2008.
2. K. Rockwood et al. A global clinical measure of fitness and frailty in elderly people. CMAJ 2005;173:489-495.

© Rockwood Version 1.2, 2009. All rights reserved.
Danish Version 1, 2020, translated by Anders Fournaise and Søren Kabell Nissen.

DALHOUSIE UNIVERSITY *Inspiring Minds*

Contact information: Anders Fournaise – anders.fournaise@rsyd.dk / Søren Kabell Nissen – sknissen@health.sdu.dk
